# The complete chloroplast genome of *Artocarpus altilis* (Moraceae) and phylogenetic relationships

**DOI:** 10.1080/23802359.2021.1945504

**Published:** 2021-07-14

**Authors:** Ueric José Borges de Souza, Raíssa Nunes dos Santos, Renisson Neponuceno de Araújo Filho, Gil Rodrigues dos Santos, Renato Almeida Sarmento, Fabien De Bellis, Fabrício Souza Campos

**Affiliations:** a Laboratory of Bioinformatics & Biotechnology, Federal University of Tocantins, Gurupi, Brazil; b Department of Forestry Engineering, Federal University of Tocantins, Gurupi, Brazil; c Laboratory of Phytopathology, Federal University of Tocantins, Gurupi, Brazil; d Post-Graduate Program in Plant Production, Federal University of Tocantins, Gurupi, Brazil; e CIRAD, UMR AGAP Institute, Montpellier, France; f UMR AGAP Institute, University of Montpellier, CIRAD, INRAE, L’Institut Agro, Montpellier, France

**Keywords:** Plastid genome, plastome, phylogenetic relationships

## Abstract

The chloroplast (cp) is an essential organelle in higher plants. The genes of the plastome are well suited to infer phylogenetic relationships among taxa. In this study, we report the assembly of the cp genome of *Artocarpus altilis* and its phylogeny among species from Moraceae family. The cp genome of *A. altilis* was 160,822 base pair (bp) in length, comprising one large single-copy region of 88,692 bp, one small single-copy region of 19,290 bp, and a pair of inverted repeat regions (IRs) of 26,420 bp. A total of 113 different genes were predicted, including 79 protein-coding genes, 30 *t*RNA genes, and four *r*RNA genes. The phylogenetic analysis of 19 species belonging to the Moraceae family confirmed the phylogenetic proximity of the genus *Artocarpus* and *Morus* and the genetic similarity of *A. camansi* and *A. altilis*.

The genus *Artocarpus* J.R. Forster and G. Forster is the most diverse of the tribe Artocarpeae and the third largest genus in the plant family Moraceae. This family includes approximately 70 species most of them native from the warm regions of South and Southeast Asia and Oceania (Zerega et al. 2010; Williams et al. 2017). Several species of *Artocarpus* are important food sources for forest fauna, and a dozen species are important crops in the regions in which they occur, such as the breadfruit (*A. altilis*), breadnut (*A. camansi* Blanco), jackfruit (*A. heterophyllus* Lam.), cempedak (*Artocarpus integer* (Thunb.) Merr.), and terap (*Artocarpus odoratissimus* Blanco) (Zerega et al. 2010). The breadfruit (*A. altilis*) is a cultigen originally derived from the wild progenitor *A. camansi*, a native species from New Guinea, where domestication was initiated (Zerega et al. 2004, 2005). The species is a staple food in tropical regions and is widely used as functional food because of the high nutritional and energy values (Gonçalves et al. 2020).

Nowadays, with the advent of next-generation sequencing technologies (NGS), the field of chloroplast (cp) genetics and genomics has expanded dramatically (Souza et al. 2019; Souza et al. 2020; Mehmood et al. 2020). At the present time, approximately 4896 complete plant cp genomes have been deposited as RefSeq in the NCBI Organelle Genome database (December 2020), although 17 of these species belong to the Moraceae family and only two to the *Artocarpus* genus. In this study, we report the complete cp genome of *A. altilis* from whole-genome sequence data and the phylogenetic analysis to evaluate the relationships among species from the Moraceae family.

Illumina paired-end sequencing data of *A. altilis* were obtained from the NCBI Sequence Read Archive (Accession no. SRR3194007; BioSample: SAMN04508170). The plant sampling, library preparation, and parameters used for high throughput sequencing are available in De Bellis et al. (2016). Briefly, fresh leaf sample of *A. altilis* cultivar ‘Novan’ was collected in Malekula Island, Vanuatu. The specimen (voucher: VUT002) was provided by Dr Roger Malapa of the Vanuatu Agricultural Research and Technical Center (VARTC). Dr Malapa passed away a few years ago. A contact could be Mrs Juliane Kaoh, in charge of the VARTC horticultural division (kaohjuliane8@gmail.com) or Jean-Pierre Labouisse (jean-pierre.labouisse@cirad.fr). The VARTC genebank is located on the Island of Santo, Vanuatu (15.453°S, 167.184°E). Total genomic DNA was extracted according to the mixed alkyl trimethylammonium bromide (MATAB) protocol described by Risterucci et al. (2000) and used to generate the library with the Nextera DNA Library Preparation Kit (Illumina, San Diego, CA) according to the manufacturer’s protocol. Paired-end sequencing was carried out at the Grand Plateau Technique Régional platform (Montpellier, France; http://www.gptr-lr-genotypage.com) on an Illumina MiSeq system using the MiSeq Reagent Kit version 3 (2 × 300 bp). The raw data (fastq files) was used to assemble the complete cp genome by using Fast-Plast pipeline version 1.2.8 with the Rosales order as the *bowtie_index* (McKain and Wilson 2017). Genome annotation was performed with Geseq (Tillich et al. 2017) and adjusted manually through comparisons with the annotations of *A. heterophyllus* (MK303549.1) and *A. camansi* (MW149075.1) using Geneious version 11.0.4 (Biomatters Ltd., Auckland, New Zealand).

The cp genome of *A. altilis* is 160,822 bp in length and presents the circular quadripartite structure typical of the angiosperms with a pair of inverted repeats regions (IRs) of 26,420 bp, separated by a large single-copy region (LSC) and a small single-copy region (SSC) of 88,692 and 19,290 bp, respectively (Supplementary Figure S1). The overall GC content (or guanine-cytosine content) was 36.0%. The cp genome of *A. altilis* was predicted to encode 113 different genes, with 79 protein-coding genes, 30 transfer RNA (*t*RNA), and four ribosomal RNA (*r*RNA) genes. Eighteen genes were duplicated completely in the IR region. Nine of the protein-coding genes and six tRNAs each contained one intron, while three genes each contained two introns. In general, the gene content, order, and organization of the *A. altilis* cp genome is highly similar to that of the closely related *A. camansi* (Souza et al. 2020) and *A. heterophyllus* (Liu et al. 2017), reported previously.

**Figure 1.**
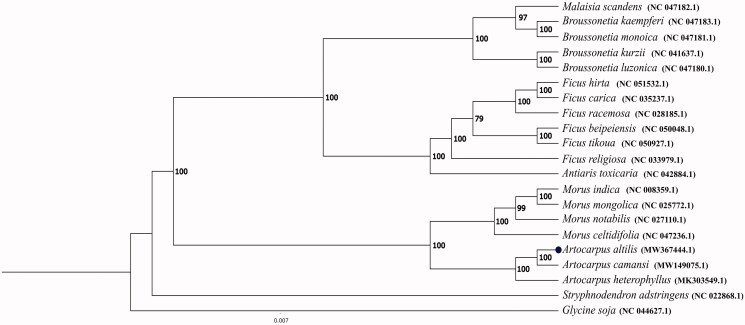
The phylogenetic tree of 19 species of the family Moraceae based on the sequences of 65 chloroplast genes. Bootstrap support values > 79% are given at the nodes.

To conduct the phylogenetic analysis, 65 protein-coding genes were recorded in 19 species from the plant family Moraceae and two species (*Glycine soja* and *Stryphnodendron adstringens*) included as outgroups. All the genes were obtained from NCBI GenBank except those of *A. altilis* (see Supplementary Table S1 for species and accession numbers). The phylogenetic tree was constructed by maximum likelihood (ML) method using RAxML version 8 (Stamatakis 2014), with 1000 bootstrap replicates (Wang et al. 2018; Lei et al. 2020). A ML analysis yielded a tree topology with 79–100% bootstrap values at each node based on GTR + G + I model (Figure 1). The phylogeny supports the close relationship between *Artocarpus* and *Morus* genus and also reinforces the genetic similarity of the plastid genomes of *A. altilis* and *A. camansi*. Besides, more recently, a phylogenetic study showed that *A. camansi* and *A. mariannensis* form a clade with *A. altilis* (Williams et al. 2017), which is not surprising, since these two species have been successfully used in breeding with *A. altilis* (Jones et al. 2013).

## Data Availability

The genome sequence data that support the findings of this study are openly available in GenBank of NCBI at (https://www.ncbi.nlm.nih.gov/) under the accession no. MW367444. The associated BioProject, SRA, and Bio-Sample numbers are PRJNA312880, SRR3194007, and SAMN04508170, respectively.
